# Sustained high prevalence of sexually transmitted infections among female sex workers in Cambodia: high turnover seriously challenges the 100% condom use programme

**DOI:** 10.1186/1471-2334-8-167

**Published:** 2008-12-12

**Authors:** Heng Sopheab, Guy Morineau, Joyce J Neal, Vonthanak Saphonn, Knut Fylkesnes

**Affiliations:** 1 National Center for HIV/AIDS, Dermatology and STDs, Cambodia; 2 Family Health International, Thailand; 3 Centers for Disease Control and Prevention, Global AIDS Program, Cambodia; 4 Centre for International Health, University of Bergen, Norway

## Abstract

**Background:**

Cambodia's 100% Condom-Use Programme (CUP), implemented nationally in 2001, requires brothel-based female sex workers (FSWs) to use condoms with all clients. In 2005, we conducted a sexually transmitted infection (STI) survey among FSWs. This paper presents the STI prevalence and related risk factors, and discusses prevalence trends in the context of the 100% CUP in Cambodia.

**Methods:**

From March-May, 1079 FSWs from eight provinces consented to participate, provided specimens for syphilis, chlamydia, and gonorrhoea testing, and were interviewed. Univariate and multivariate logistic regression analysis was used to determine factors associated with STIs. STI prevalence was compared with data from the 1996 and 2001 STI surveys.

**Results:**

Most FSWs were young (55% aged 15–24) and new to sex work (60% had worked 12 ≤ months). Consistent condom use with clients was reported by 80% of FSWs, but only 38% of FSWs always used condoms with sweethearts or casual partners. Being new to sex work was the only factor significantly associated with "any STI" (OR = 2.1). Prevalence of syphiliwas 2.3%; chlamydia, 14.4%; gonorrhoea, 13.0%; and any STI, 24.4%. Prevalence of each STI in 2005 was significantly lower than in 1996, but essentially the same as prevalence observed in 2001.

**Conclusion:**

New FSWs were found to have substantially higher prevalence than those with longer experience. The percent of FSWs who used condoms consistently was high with clients but remained low with non-paying sex partners. Because of the high turnover of FSWs, the prevention needs of new FSWs should be ascertained and addressed. Despite 100% CUP implementation, the prevalence of STIs among FSWs was the same in 2005 as it was in 2001. Limited coverage and weak implementation capacity of the programme along with questionable quality of the STI services are likely to have contributed to the sustained high prevalence. The programme should be carefully reviewed in terms of intensity, quality and coverage.

## Background

Sexually transmitted infections (STIs) remain major causes of reproductive morbidity and mortality in developing countries; and their high prevalence facilitate HIV transmission [1]. STIs are usually concentrated in core groups such as female sex workers (FSWs) characterized by a high number of partners and poor healthcare seeking behavior [2]. The prevalence of gonorrhoea and/or chlamydia among FSWs was about 20% in 2003 in some Vietnamese provinces that share a border with Cambodia (with geographical variation ranging from 11.3% to 32.7%) [3], and 18% in 2004 in Laos (with provincial variation ranging from 12.3% to 21.9%) [4]. In Chiang Mai, Thailand, the 2004 prevalence of gonorrhoea and chlamydia among FSWs was 14% and 17% respectively [5], whereas the prevalence of gonorrhoea or chlamydia in China's Yunnan province was 24.6%.

In Cambodia, the first STI prevalence study conducted in 1996 found a very high prevalence of STIs among FSWs; 38.7% of these women had gonorrhoea and/or chlamydia and 13.8% had active syphilis [6]. Consequently, programmes focusing on FSWs have been widely implemented. One such intervention is the 100% condom use programme (CUP), which was based on the model successfully implemented in Thailand in 1989. The 100% CUP is a multi-sectoral approach involving local authorities, health staff, police, brothel owners, sex workers, outreach and peer educators, with the aim of enforcing condom use in all brothels and sex-related establishments [7]. This programme encourages FSWs to use condoms consistently with clients and regularly attend STI check-ups at assigned clinics. The strategy is based on promotion of systematic use of condoms in all brothels and entertainment establishments, and referral to free-of-charge STI clinics by stakeholders including peer-educators, establishment managers, health staff, and the local police. A Condom Use Working Group, consisting of police, local authorities, and health staff is set up to monitor, register and refer FSWs to assigned STI clinics and enforce programme implementation [8]. A brothel would be temporarily closed for one month if women in that specific brothel were found STIs 3 consecutive times during the regular STI check-up at the assigned clinic, but women would not receive any penalties. This programme was piloted in 1998 in Sihanouk Ville, a province characterized by a high prevalence of HIV infection (52% in 1996) among brothel-based FSWs. A programme evaluation, conducted eighteen months after the implementation, found a substantial reduction in STI incidence at the clinic designated for use by FSWs. From 1998 to 2000, syphilis incidence declined from 9.0% to 1.8% and trichomoniasis incidence declined from 2.0% to 0.9%. Almost all (95%) FSWs attended the regular monthly check-up at the STI clinics, and more than 90% reported always using condoms with clients. Based on this success, the Prime Minister supported the 100% condom use policy for national use and the programme was implemented nationwide in 2001 [9].

Compared to HIV that is a life long infection, curable bacterial STIs are biological markers that are more likely to reflect recent risk behaviour. While high STI prevalence indicates frequent risky sexual practice and a poor provision or uptake of services, low STI prevalence reflects the improvement in provision of care services or change in risk behaviours [10]. Compared with the 1996 prevalence estimates, the 2001 Cambodia STI Surveillance Survey (SSS) results indicated a significant decline of major STIs among FSWs; gonorrhoea decreased from 24.0% to 14.2%; chlamydia from 23.3% to 12.1%; and syphilis from 14.0% to 5.7% [11]. The high turnover of sex workers observed in the 2003 Behavioural Surveillance Survey (BSS) (i.e., 50% of FSWs reported that they had been selling sex for less than one year) [12] is a major concern because it renews the pool of FSWs who have not yet been exposed to sexual health promotion messages or monthly STI exams. In this paper, we examine the relationship between duration of sex work and STI prevalence among FSWs. We then discuss the trends in prevalence of STIs from 1996 to 2005 with regards to the effectiveness of the 100% CUP in Cambodia.

## Methods

### Data collection

This report is based on data from Cambodia's national SSS 2005 conducted among brothel-based FSWs from March to August 2005 in the 8 capital cities of the following provinces: Phnom Penh, Kampong Cham, Prey Veng, Battambang, Banteay Meanchey, Siem Reap, Koh Kong and Sihanouk Ville. Brothel-based FSWs were defined as women mainly working at the brothels in the red light areas who have no other employment other than selling sex to clients. Prior to data collection, the National Center for HIV/AIDS, STI and Dermatology (NCHADS) and Provincial AIDS Offices together with local NGOs conducted mappings of all known brothels located in all survey provinces, which served as sampling frames. All women working in selected establishments and not menstruating at time of interview were eligible. Participants were selected through two-stage cluster sampling. At the first stage, brothels were selected with equal probability. At the second stage all women working in selected brothels were invited to visit the nearby local STI clinics or a mobile clinic established in the vicinity. Women who provided informed consent were interviewed by trained staff about socio-demographic information, sexual risk behaviours, self-perception of risk for STIs, STI history and STI-related health seeking behaviours. Nurses collected venous blood (7 ml) and provided detailed instruction for women to self-collect vaginal swabs.

### Specimen storage and transport, and laboratory method and quality control

Whole blood was centrifuged, aliquoted and stored at 4°C at each survey site before being transported to Phnom Penh within 48 hours. All specimens were stored at 4–6°C at the National Clinic for Dermatology and STD (NCDS) laboratory until being tested. Swabs were tested within 4 days of collection. Serum were tested for syphilis using Determine Syphilis TP (Abbott Diagnostics) which is highly sensitive (92.3%) and specific (100%) [13]. This test was done in the field to provide result on-the-spot for offering treatment for women only. Each participant whose syphilis test was positive was offered treatment according to national guidelines. Moreover, all consenting FSWs were offered single-dose presumptive treatment for gonorrhea and chlamydia (medication and dosage consistent with national guidelines). Swabs were tested for *Neisseria gonorrhoeae *(NG) and *Chlamydia trachomatis *(CT) by the NCDS laboratory using BD Probe Tec™ strand displacement amplification assay. Serum specimens were tested for syphilis with rapid plasma reagin (RPR) and RPR-reactive specimens were confirmed by *Treponema pallidum *Particle Agglutination (TPPA). It meant that syphilis was defined as those who were both RPR and TPPA positive. Laboratory methods for identifying NG and CT and confirming RPR-reactive specimens were different from those used in the 1996 and 2001 surveys. CT and NG were tested using Ligase Chain Reaction in 1999 whereas Polymerase Chain Reaction was used in 2001. In the two previous rounds of survey, syphilis was tested using RPR and TPHA. Finally all NG- and CT-positive and 10% of negative specimens were retested by real-time Polymerase Chain Reaction by US CDC (Atlanta), considered as the gold standard for comparison.

### Statistical analysis

The data were coded and entered into EpiData 3 (Odense, Denmark) and analysis was performed using STATA 10 using the survey commands (Stata Corporation, Texas, USA). The sampling design is self-weighted within provinces [14], but data were weighted to account for differences in the reported numbers of FSWs between provinces. Analysis was performed using survey commands taking into account sampling weight, cluster effect and stratification by province. Descriptive statistics were used to describe variables in terms of frequency, mean and median. Prevalence of NG, CT, syphilis, and "any STI" were estimated with 95% confidence intervals that account for the complex survey design, including provincial sampling weights and primary sampling units or clusters (brothels). Stata software uses the cluster information to calculate design effects and adjusts the standard errors before conducting statistical tests. Socio-demographic characteristics and risk behaviors were cross-tabulated with the variable "any STI," which included any of the following: gonorrhoea, chlamydia, or syphilis. Chi-square test, univariate, and multivariate logistic regression were used to determine factors independently associated with STI prevalence. After completion of the univariate analysis, candidate variables were selected for inclusion in the final regression model based on having a p-value < 0.20 [15] in the univariate analysis or if they were thought to be associated with having an STI. Associations with a p-value < 0.05 were considered statistically significant. Colinearity between variables in the final model was not observed. Differences in STI prevalence were examined based on comparison of data from this survey with epidemiological data from 1996 [6] and SSS 2001. To ensure comparability over time, the comparative analysis was restricted to four provinces covered by all three surveys (Banteay Meanchey, Battambang, Sihanouk Ville, and Phnom Penh) and Kampong Cham province, which was covered by the 2001 and 2005 surveys.

The SSS 2005 protocol was approved by the Cambodia National Ethics Committee, the Institutional Review Board of Family Health International, and the Centers for Disease Control and Prevention.

## Results

### Socio-demographic, risk behaviours, risk self-perception and STI-related health seeking behaviours

Of 1081 FSWs invited to participate in SSS 2005, 1079 consented (99.8% acceptance). Blood was collected from 1079 participants, and 1063 provided vaginal swabs. Mean age of participants was 25 years (Median = 23 years) and 55.4% were younger than 25 years old. Mean years of schooling was 2.4 (Median = 2 years); 44% had never attended school, whereas 9.2% had attended secondary school (≥ 7 years). The women were highly mobile, i.e., about half of them reported living in the current provinces or cities for less than 12 months, 76% of whom had lived in at least two provinces in the past year.

The duration of sex work ranged from 1 month to 12 years, and 60% were "new FSWs" (having sold sex for 12 months or less). More than half of the women had experienced at least one abortion. Although no significant differences between new and longer-working FSWs were observed regarding report of abortion and places where women sought care for their last abortion, a larger proportion of new FSWs reported their last abortion in the past 6 months (63.2% vs. 50.1%) (table 1). Sweetheart relationships (defined in Cambodia as noncommercial, non-marital sexual relationship that possess a certain degree of affection and trust from at least one partner) in the past 12 months were reported by 58% of FSWs. Among these FSWs, 98% reported having sexual relations with their sweetheart in the past three months. The percent of FSWs who reported consistent condom use in the past month with sweethearts (25%) and casual partners (i.e., a non-paying partner other than the sweetheart with whom women neither are married nor living) (34%) was low. In contrast, 80% of FSWs reported always using condoms in the past week with clients. This high level of consistent condom use was achieved despite the fact that 67% of FSWs reported having been forced or convinced not to use a condom by clients in the past week. About one third of FSWs reported intercourse with clients during their menstruation in the past month. No significant differences between new and longer-working FSWs were found in terms of condom use with clients, sweethearts, casual partners, and number of clients per day.

**Table 1:** Sexual behaviour, self-perception of risk, and STI-related health seeking behaviour among FSW, by duration of sex work

**Characteristics**	**Worked as FSW ≤ 12 months** **n = 674**	**Worked as FSW > 12 months** **n = 404**	Total* n = 1078	**Chi square P value**
	**% (n)**	**% (n)**	**% (n)**	
Report of having at least 1 abortion	50.0 (248)	59.5 (212)	54.0 (460)	0.088
Most recent abortion in the last 6 months†	63.2 (150)	50.1 (105)	58.5 (255)	0.151
Place women sought last abortion†				
Private doctor/midwife/nurse	87.1 (202)	85.9 (181)	86.5 (383)	0.209
Traditional practitioner	8.8 (14)	5.3 (7)	7.1 (21)	
Other (mostly public and NGOs clinics)	4.1 (18)	8.9 (18)	6.4 (36)	
Report of having sweetheart (SH) in the past 12 months**	54.0 (318)	63.4 (197)	58.0 (515)	0.118
Report of having sex with SH in the past 3 months***	97.3 (281)	98.9 (173)	98.0 (454)	0.148
Report of having >3 clients in the last working day	42.7 (233)	38.4 (139)	40.9 (372)	0.422
Always used condoms with clients in the past week	81.0 (582)	80.0 (356)	80.2 (938)	0.596
Always used condoms with sweetheart in the past month***	29.4 (110)	19.6 (59)	25.0 (169)	0.069
Always used condoms with casual partner in the past month† †	37.4 (49)	29.9 (41)	34.1 (90)	0.266
Continued having sex during menstruation in the past month	29.8 (166)	33.2 (126)	31.2 (292)	0.533
Ever forced/convinced by client not to use condom in the past month	68.3 (302)	65.0 (166)	67.0 (468)	0.584
Self-perceived risk of getting sexual diseases compared with other Cambodian women				
Much higher	19.0 (214)	24.9 (136)	21.5 (350)	**0.016**
A little bit higher	24.1 (114)	19.6 (57)	22.2 (171)	
Similar risk	18.2 (85)	28.8 (63)	22.7 (148)	
Lower risk	38.7 (152)	26.7 (93)	33.6 (245)	
Believed they had an STI in the past year	37.5 (254)	61.4 (211)	47.1 (465)	**<0.001**
Places where treatment was sought for the last STI‡				
Public hospital or STD clinic	68.4 (198)	62.5 (178)	65.2 (376)	0.558
Private hospital/medical staff	2.1 (14)	4.0 (16)	3.1 (30)	
Pharmacy	7.9 (23)	8.0 (19)	7.9 (42)	
Non-governmental organisation clinic	21.0 (15)	22.8 (17)	22.0 (32)	
Did not stop having sex during the last STI episode‡	53.0 (131)	40.6 (107)	46.5 (238)	0.289

*Total does not add to 1079 because of missing information on duration of sex work

† Among those who reported having an abortion

** SH: Sweetheart, non-marital and non-commercial sex partner

*** Among those who reported having sweehearts

† † Non-paying partner with whom women were neither married nor living and who was not their sweetheart

‡ Among those who reported having an STI

Only 22% of FSWs perceived themselves to be at much higher risk than other Cambodian women of becoming infected with an STI, and the longer-working FSWs were somewhat more likely than the new FSWs to perceive themselves as being at much higher risk (24.9% vs. 19.0%, p = 0.016). New FSWs were less likely to report having experienced STI symptoms in the past 12 months compared with their longer-working peers (37.5% vs. 61.4%, p < 0.001); no difference between the two groups was observed regarding places where they received care for their last STI episode. New FSWs, however, tended to be more likely than the longer-working FSWs to have continued having sex with clients during the last STI episode (table 1).

### STI prevalence

Estimated prevalence of gonorrhoea, chlamydia, and syphilis is summarized in table 2. A total of 13% had gonorrhoea, 14% had chlamydia, and 2% had been infected with syphilis. All together, 24.4% had at least one STI. Prevalence of gonorrhoea, chlamydia, and any STI among the new FSWs was higher than among the longer working FSWs: prevalence of gonorrhoea was 16.5% vs. 7.7% (p = 0.010); chlamydia, 17.9% vs. 9.0% (p = 0.003); and any STI, 30.0% vs. 15.8% (p < 0.001). Prevalence of syphilis, however, was lower among the new FSWs than among their longer working peers (1.3% vs. 3.8%, p = 0.011). Quality control retesting of vaginal swab specimens showed the test used was 96.3% sensitive and 97.7% specific for *N. gonorrhoeae*, and 92.6% sensitive and 95.7% specific for *C. trachomatis*. Predictive value of a positive test was 97.0% for *N. gonorrhoeae *and 95.2% for *C. trachomatis*.

**Table 2:** STI prevalence among FSWs, by duration of sex work

**STI prevalence**	**Worked as FSW ≥ 12 months**		**Worked as FSW> 12 months**		**Chi square P Value**	**Total**	
	n/N*	% (95% CI)**	**n/N**	% (95% CI)**		**n/N**	% (95% CI)**
Syphilis, (RPR/TPPA)	19/674	1.3 (0.7–2.3)	15/404	3.8 (1.9–7.3)	0.011	34/1078	2.3 (1.3–3.9)
Chlamydia	109/668	17.9 (13.9–22.9)	42/395	9.0 (5.5–14.3)	0.003	151/1063	14.4 (11.0–18.6)
Gonorrhoea	82/666	16.5 (11.4–23.4)	40/395	7.7 (4.3–12.9)	0.01	122/1061	13.0 (9.2–18.0)
Any STI***	172/666	30.0 (23.8–37.1)	77/395	15.8 (11.4–21.5)	<0.001	249/1061	24.4 (19.8–29.7)

*N = number of specimens tested; number varied because among FSWs for whom duration of sex work was known, 15 FSWs did not submit swabs and 2 swabs were not tested for gonorrhoea

**CI = confidence interval

**Any STI = number of women with at least one of the bacterial STIs

### Factors associated with having "any STI"

Results of univariate and multivariate analyses for the risk of having any STI are presented in table 3. Factors significantly associated with "any STI" in the univariate analysis were: living in the current city for ≤ one year (odd ratio [OR] = 1.86, 95% confidence interval [CI]: 1.22, 2.81) and being a new FSW (OR = 2.29, 95% CI: 1.44, 3.65). Variables included in the final multivariate model were age group, education, abortion, duration of sex work, number of clients in the last working day, sex during menstruation, and having an STI in the past year. After controlling for other variables, however, only duration of sex work was associated with "any STI"; new FSWs were 2.1 (95% CI: 1.21, 3.78) times more likely to be infected than those who had been working longer. Less than 6 years of education (OR = 2.36, 95% CI: 0.98, 5.69), and sex during menses (OR = 1.57, 95% CI: 0.98, 2.53) were marginally associated with the risk of carrying any STI.

**Table 3:** Factors associated with STI in univariate and multivariate logistic regression

**Characteristics**	Total, N = 1062*			**Total, N = 1054**
		
	**No. (% STI positive)**	**OR (95% CI)**	**P Value**	AOR (95% CI)**
Age group (years)				
15–24	658 (25.0)	Referent		Referent
25–29	294 (23.6)	0.92 (0.55–1.56)	0.450	0.84 (0.48–1.46)
≥ 30	110 (23.7)	0.93 (0.50–1.81)		1.20 (0.54–2.60)
Education level				
0–5 years	889 (26.2)	2.10 (0.94–4.62)	0.063	2.36 (0.98–5.69)
≥ 6 years	162 (14.5)	Referent		Referent
Duration living in the current city				
≤ 12 months	628 (30.0)	1.86 (1.22–2.81)	0.004	1.36 (0.86–2.16)
> 12 months	434 (18.8)	Referent		Referent
History of ever having had an abortion				
No abortion	606 (27.4)	Referent		Referent
Any abortion	455 (22.1)	0.75 (0.50–1.13)	0.168	0.76 (0.47–1.21)
Duration of sex work				
≤ 12 months (new FSW)	666 (30.0)	2.29 (1.44–3.65)	<0.001	2.14 (1.21–3.78)
> 12 months (longer working FSW)	395 (15.8)	Referent		Referent
Reported having sweetheart in the past 12 months				
No sweetheart	556 (27.3)	Referent		
Any sweetheart	505 (21.8)	0.74 (0.42–1.33)	0.314	-
Number of clients in last working day				
≤ 3 clients	691 (22.3)	Referent		
> 3 clients	368 (27.5)	1.32 (0.84–2.10)	0.231	-
Condom use with clients in the past week				
Always use condom	924 (23.0)	Referent		Referent
Not always use condom	114 (30.3)	1.45 (0.83–2.53)	0.183	1.37 (0.77–2.45)
Working during menstruation				-
Continued working	289 (30.4)	1.56 (0.97–2.50)	0.063	1.57 (0.98–2.53)
Stop working ≥ 1 day	759 (21.9)	Referent		Referent
Convinced/forced by clients not to use condom				
Never convinced/forced	552 (21.1)	Referent		
Ever convinced/forced	459 (26.3)	1.34 (0.83–2.17)	0.231	-
Self-report of STI in the past year				
No	603 (21.5)	Referent		Referent
Yes	457 (27.8)	1.40 (0.82–2.43)	0.216	1.70 (0.95–3.00)

*Total does not equal 1062 for all variables because of missing information

** Adjusted odds ratio and 95% confidence interval

### STI prevalence trends

Table 4 presents the STI data among FSWs from 1996, 2001 and 2005. Overall, the STI prevalence declined from 1996 to 2001 and was stable from 2001 to 2005. Syphilis prevalence declined from 13.8% in 1996 to 3.7% in 2001 but had not changed (3.6%) in 2005; gonorrhoea prevalence declined from 23.2% in 1996 to 13.3% in 2001 but stayed at 13.0% in 2005; chlamydia prevalence dropped from 22.5% in 1996 to 13.1% in 2001 but remained at 14.3% in 2005.

**Table 4:** STI prevalence in five provincial towns^a ^in Cambodia, by year, 1996–2005

**STI testing**	**1996**^**b**^	**2001**	**2005**
			
	**N = 437** **% (95% CI^**d**^)**	**N = 406** **% (95% CI)**	**N = 797^**c **^** **% (95% CI)**
Syphilis (RPR/TPHA)	13.8 (10.6–17.0)	3.7 (1.9–5.5)	3.6 (2.3–4.9)
Gonorrhoea	23.2 (19.2–27.2)	13.3 (10.0–16.6)	13.0 (10.7–15.4)
Chlamydia	22.5 (18.6–26.4)	13.1 (9.8–16.4)	14.3 (11.9–16.8)
Gonorrhoea/chlamydia	38.7 (34.1–43.3)	22.7 (18.6–26.8)	22.5 (19.6–25.4)

a. Towns included in the comparison by survey year were Phnom Penh and the capital cities of Banteay Meanchey, Battambang, Kampong Cham, and Sihanouk Ville provinces. Data from Kampong Cham were not available for 1996

b. Ryan et al., Lancet 1998

c. The total number of specimens tested for gonorrhoea and chlamydia was 784

d. CI = confidence interval

## Discussion

As revealed in the 2005 survey, women who were new to sex work contributed substantially to the sustained high prevalence of STIs, i.e., about twice the prevalence of those with longer sex-work experience. The 2005 data did not provide any behavioural explanations to these observed differences in STI prevalence by duration of sex work. Indeed, the number of clients per day, intercourse with clients during menstruation, STI-related treatment seeking behaviours, consistent condom use with clients, sweethearts or other non-paying sex partners and to what extent the FSWs had been forced or convinced not to use condoms by clients did not differ by duration of sex work. Among FSWs new to sex work, 59% were aged 15–24, whereas 50% of longer-working FSWs were aged 15–24. Because of their slightly younger age, FSWs new to sex work may have been more biologically vulnerable to acquiring STIs than those who had been working longer [3].

To date, the association between STI prevalence and recent entry into the sex trade has not been studied in Cambodia. The association of being new to sex work and having an STI may suggest higher prevalence of high risk behaviour possibly related to the lack of exposure to prevention information, unawareness of STI services, limited access to outreach programmes, and less skill and limited experience in negotiating safer sex with clients. However, our analysis did not reveal any significant differences between new and longer-working FSWs in terms of socio-demographic characteristics and risk behaviours that might explain the significant difference in prevalence of STIs. Therefore, a biological difference between new FSWs and longer-working FSWs is one possible explanation. A similar finding was reported in a report from Vietnam in which the authors suggested that the higher prevalence of STIs among newer FSWs may be attributed to a lack of immunity to chlamydia compared with longer-working FSWs [3]. Other possibilities to consider are that new FSWs may have clients who are different from or at higher risk than clients of other FSWs and that new FSWs may have casual partners or sweethearts that are at higher risk. These issues should be addressed in future surveys or studies.

Reproductive health issues are serious concerns for FSWs as suggested by the large proportion who reported having an abortion in the past 6 months, especially for the newer FSWs. Abortions indicate unwanted pregnancy and unprotected sex which may be the result of less consistent use of condoms with sweethearts or casual partners. Providing sex services to clients during menses (~30%) is likely to put women at higher risk of acquiring STI/HIV infection due to vaginal fragility. A number of studies found an association between sex during menses and increased risk of gonorrhoea and chlamydia [16,17]. Therefore, outreach and peer education programmes for FSWs should address all these reproductive health issues. A low level of education appeared to be a risk factor for STIs. Women with more education may be in a better position to access educational information due to their ability to read and better understand preventive messages. However, many studies show no correlation between level of education and STIs among FSWs [3,18,19].

The majority (80%) of FSWs reported always using condoms in the past week with clients, which seems discordant with the fact that 67% of FSWs reported having been forced or convinced not to use a condom by clients in the past week. Reasons for these seemingly discordant results may be related to interpretation of the question. Some FSWs may have misinterpreted the question about whether a client forced them not to use condoms to mean, "Has a client *tried *to force you not to use condoms?" Another explanation may be that FSWs who had been forced not to use condoms may not have counted such involuntary episodes as a lapse in condom use and reported that they used condoms consistently in the past week.

Problems with this question were not revealed by pilot testing. This issue should be addressed by future surveys or studies. Although a small proportion of FSWs reported not always using condoms with clients, the fact that a large proportion had unprotected sex with their sweethearts may partially explain the sustained high STI prevalence in 2005. If sweethearts become infected and do not receive treatment they may serve as reservoirs for re-infection of FSWs who were successfully treated as part of the 100% CUP. Over the past 10 years, the proportion of FSWs in Cambodia reporting consistent condom use with sweethearts has never reached 60% [12] although repeated efforts have been made to address this issue in outreach and peer education programmes. However, it is unknown how well the training and outreach and peer education activities have been translated into action that may impact behaviour with non-paying partners. Concerned about the quality of the outreach and peer education programme, NCHADS, in collaboration with many stakeholders including the National AIDS Authority, Ministry of Women Affairs, and nongovernmental organizations, have recently revised the standard operating procedures for the outreach/peer education programme and 100% CUP for sex workers, in an effort to make it more sustainable and efficient and less subject to service disruption [20].

Data from three STI prevalence surveys conducted in 1996, 2001, and 2005 showed a marked decline in STI prevalence from 1996 to 2001, but no appreciable decline was observed from 2001 to 2005. It is widely accepted that the 100% CUP is an effective measure in the prevention and control of HIV infection among sex workers because of the reduction of STI incidence and prevalence and the reinforcement of behaviour change if properly implemented [7,8]. Nevertheless, the lack of further decline in prevalence of STIs from 2001 to 2005 may indicate some limitations in the implementations of the programme. The temporary disruption of the preventive programme in 2004 and 2005 because of central management staffing shortages may have affected programme effectiveness. Moreover, provincial staff turnover, and delayed or limited funding at the provincial level may have affected the implementation and overall quality of the programme. These weaknesses were documented in a 100% CUP supervision report (*Personal communication with NCHADS Behaviour Change Communications Unit, 2005*). Furthermore, the contradictory attitudes of the government toward sex work (e.g. sometimes endorsing programmes to make sex work safe, sometimes closing sex work establishments) make it difficult for sex workers, and brothel owners and managers to understand and trust the government's intentions and motives [21]. Finally, some brothel owners and FSWs may be uncomfortable collaborating honestly and participating in the 100% CUP because of the involvement of local police in the Condom Use Working Group. On average, about 60% of FSWs visited STI clinics each month in 2005, indicating poor coverage of the 100% CUP.

In addition to quality of interventions, mobility of sex worker and coverage of the programme are main concerns with regards to preventive interventions. Many studies have reported that FSWs often move from place to place in order to maximise income. As they age, some move to more rural settings where competition for clients is less [22,23]. As a result, they may have less access to the outreach/peer education programme, condoms, or STI care services that are mostly concentrated in provincial towns or urban settings. Therefore, coverage of the preventive programme should be considered when interpreting these data. FSWs also move from one type of sex work to another – i.e., from brothel-based to non-brothel-based and vice versa [12] – which disrupts the continuity of prevention programme exposure. This instability is aggravated by the frequent police closure of brothels, which leads women moving to other forms of sex work or to other provinces. For example, 25% of brothel-based FSWs reported previous employment as karaoke workers and 50% of beer promoters reported formerly working in brothels or as dancing girls or karaoke workers. Non-brothel-based FSWs represent about 60% of the sex worker population in Cambodia [24] and because many deny engaging in sex work they have not been significantly covered as a group by the 100% CUP. A study in Battambang province in north-western Cambodia, found a high STI prevalence among beer promoters, suggesting limited effectiveness of the existing STI programme and limited targeted intervention for non-brothel-based FSWs [25]. Therefore, the effectiveness of the 100% CUP may be limited because of its low overall coverage of the sex industry as a result of targeting only brothel-based FSWs. The 100% CUP works well if implementation is rigorously conducted with high coverage and sufficient intensity as shown in Thailand in the late 1989 and in Sihanouk Ville, Cambodia in late 1998 [7,9].

Our last concern is about the quality of STI services in terms of appropriate diagnosis and treatment of cervical and vaginal infections commonly found among FSWs. Although health care services in developing countries are generally recognized to be poor, the quality of STI care is especially poor for FSWs, a stigmatized group which faces discrimination in many societies. A study in Abidjan, Ivory Coast, showed that the quality of STI services in health facilities visited by FSWs was poor; for example, knowledge among health personnel about the correct treatment of selected STIs was very low in most settings [26]. So far, little is known about the quality of the STI services in Cambodia. Therefore, immediate actions should be taken to evaluate the quality of services and perform a needs assessment. A refresher or in-service training should be provided for staff, steps to reduce attrition of staff should be implemented, technical support and supervision should be increased at the provincial level, and availability of drugs should be ensured.

A number of factors should be taken into account when interpreting comparisons of data by survey year. First, each of the three surveys employed different laboratory techniques and data collection methods. Because of variable test performance (sensitivity and specificity) different test methodologies may have a dramatic effect on the measured prevalence of STI pathogens [27]. In 1996, researchers used ligase chain reaction (LCR) to test urine specimens for CT and NG nucleic acid. Since this technique has a lower sensitivity than tests performed on vaginal swabs in subsequent studies, prevalence may have been underestimated in 1996. Subsequent STI surveys used the polymerase chain reaction (PCR) in 2001 and the strand displacement assay in 2005. Although the techniques were different, all the tests used amplification methods that target nucleic acid sequences and have comparable performance characteristics as reported by test manufacturers: high sensitivity (99%–100%) and specificity (98%–100%) [28]. In 2001, vaginal swabs were collected by medical staff, whereas swabs were self-collected in 2005. However, many studies have confirmed the similarity of the two methods in terms of sensitivity and specificity [29]. Second, varying sampling methods were used. While convenience sampling was used in 1996, the 2001 and 2005 surveys randomly selected participants using cluster sampling. Difference in sampling raises concern about comparing results from the populations sampled in 1996 with those from FSWS sampled in 2001 and 2005. On one hand, convenience sampling in 1996 might have resulted in overestimating the true prevalence since FSWs visiting STI clinics at that time-i.e., those with STI symptoms – may have been more likely to participate in the survey. On the other hand, using LCR to test urine (and not vaginal swabs) may have underestimated CT and NG prevalence in 1996. These biases are in opposite directions, and the magnitude and direction of the resulting bias are difficult to quantify. The combination STIs of short duration of infection (GC and CT) and long duration (TP) may affect the strength of association of covariates with having "any STI." However, given the low prevalence of syphilis, the relatively young age of the women and their short duration of sex work, this effect was probably minimal. Lastly, because most FSWs were familiar with the 100% CUP, social desirability bias may have resulted in FSWs underreporting the frequency of unprotected sex.

## Conclusion

STI prevalence among new FSWs was higher than among longer-working FSWs. Because of the high turnover of FSWs, the prevention needs of new FSWs, which may be different than those of longer-working FSWS, should be ascertained and addressed. Despite the implementation of a nationwide 100% CUP, the prevalence of STIs among FSWs in 2005 was comparable to 2001 estimates. The large proportion of FSWs who reported having unprotected sex with non-commercial partners, limited coverage and weaknesses in implementation of the 100% CUP, in addition to questionable quality of STI care services are likely to have contributed to the sustained high prevalence of STIs among FSWs in Cambodia. The 100% CUP should be carefully evaluated, particularly in terms of human resource capacity, sustainable intensity, quality, and coverage. Moreover, the outreach and peer education programmes should include a subcomponent which specifically targets new FSWs in terms of providing preventive messages and support services.

## Competing interests

The authors declare that they have no competing interests.

## Authors' contributions

HS, GM, JN and VS conceived and designed the study. KF provided conceptual ideas in drafting the paper. HS wrote the first draft of the paper and other coauthors contributed to the final draft. HS and GM were responsible for conducting the study and managing the data. HS and KF conducted the statistical analyses and the interpretation of data. Others participated in the data analysis and data interpretation. All authors read and approved the final manuscript.

## Pre-publication history

The pre-publication history for this paper can be accessed here:


